# The Effects of the Mamanet Cachibol League Intervention Program on Perceived Health Status, Mental Health, and Healthy Lifestyle Among Arab Women

**DOI:** 10.3390/healthcare13020169

**Published:** 2025-01-16

**Authors:** Karin Eines, Riki Tesler, Ruth Birk, Ariela Giladi, Ayelet Dunsky, Nada Alian, Limor Gonen, Sharon Barak

**Affiliations:** 1 Department of Health System Management, Faculty of Health Science, Ariel University, Ariel 40700, Israel; karin.mental@gmail.com; 2 Department of Nutrition, Faculty of Health Science, Ariel University, Ariel 40700, Israel; 3 Department of Education, Faculty of Social Sciences, Ariel University, Ariel 40700, Israel; arielagiladi@gmail.com; 4 Department of Sport, Wingate Institution, Netanya 4290200, Israel; ayelet@wincol.ac.il; 5 Ministry of Health, Tel Aviv 6473912, Israel; nada.alian1@moh.gov.il; 6 Department of Economic & Business Administration, Faculty of Social Sciences, Ariel University, Ariel 407000, Israel; limorg@ariel.ac.il; 7 Department of Nursing, Faculty of Health Science, Ariel University, Ariel 40700, Israel; sharoni.baraki@gmail.com

**Keywords:** community sports, subjective happiness, social capital, healthy lifestyle, depression symptoms

## Abstract

**Background:** The Israeli Mamanet Cachibol League (MCL) is a community-oriented athletic program serving mothers through non-competitive recreational sports participation. This study aimed to assess the effects of the MCL on perceived health status, mental health (happiness, depression, social capital), and healthy lifestyle behaviors (physical activity and nutrition). **Methods:** This is an experimental study with a sample of 231 women (174 in the experimental group and 57 in the control). Participants completed questionnaires in November 2023 (T1) and then in August 2024 (T2). The questionnaire included questions on sociodemographic characteristics, perceived health status, mental health (happiness, depression, social capital), and healthy lifestyle behaviors (physical activity and nutrition). **Results:** At T1, the MCL participants reported better mental health (higher subjective happiness and social capital and lower depressive symptoms) than the control group. Over time, participation in the MCL led to significant improvements in mental health (reductions in depression and increases in subjective happiness and social capital). The participants showed substantial improvements in healthy lifestyle behaviors, with moderate effect sizes (effects size > 0.5) observed across these areas. Sociodemographic factors influenced the outcomes, with variations in health perception and physical activity linked to marital status and education level. **Conclusions:** Participation in the MCL program was associated with better mental health at baseline and significantly improved over time compared to the control group. The MCL participants also showed gains in healthy lifestyle behaviors, highlighting the importance of tailored interventions.

## 1. Introduction

Participation in sports holds transformative potential for women’s health and well-being, particularly within minority communities [1]. Regular engagement in sports activities has been shown to significantly improve both physical and mental health outcomes, supporting overall well-being and fostering social connections [2,3]. This is especially relevant for Arab women, who often face unique cultural and social barriers to sports participation [4,5]. Recent epidemiological studies have revealed that approximately 65–75% of Arab women across various regions are physically inactive, significantly higher than the global average of 32% for women [6,7,8]. This sedentary lifestyle has been associated with high hypertension [9] and obesity rates within this population. The prevalence of obesity among Arab women has reached alarming levels, with recent studies indicating rates as high as 75% across different Arab regions [10]. This health crisis is particularly evident in Gulf Cooperation Council countries, where obesity rates among women exceed 50%, significantly higher than global averages [11].

Physical activity through sports provides numerous health benefits, including reduced obesity risk [12,13], especially through structured community programs [11,13,14,15]. Physical activity also enhances immune system function and lowers the chances of chronic illnesses like heart disease and diabetes [16,17]. Beyond physical health, sports serve as a critical space for social interaction, fostering camaraderie and support networks that enhance mental and emotional well-being [4,5,6,18]. These networks contribute to a greater sense of belonging and community, especially in societies where women may face restrictive cultural norms [19,20]. Therefore, sedentary behavior among Arab women is a pressing public health issue [1,4,21], and previous studies have underscored the critical need for tailored intervention strategies that address both physical activity and dietary habits. Public health interventions can significantly reduce the prevalence of sedentary behavior and its associated health consequences in this population by targeting these key factors and accounting for sociodemographic disparities.

Despite the well-documented benefits of structured community programs on health, participation in physical activity and sports among Arab women remains low, especially in traditional communities. Women’s participation in physical activity and sports is influenced by various sociodemographic factors such as socioeconomic status, age [22,23], and family responsibilities, which often shape their level of engagement and access to sports opportunities [24,25]. More specifically, women from different socioeconomic backgrounds may experience varying levels of access to resources such as training facilities and time for leisure activities [24,25,26]. Cultural expectations and traditions also heavily influence women’s participation in sports, particularly in more conservative societies where gender roles and societal norms may discourage women from engaging in physical activities [27]. According to the Israeli Ministry of Health, 42% of Arab women participate in regular leisure physical activity, compared to 59% of Jewish women.

The Israeli Mamanet Cachibol League (MCL) serves as an exemplary model of a community-based sports program that promotes inclusivity and physical activity. Founded in 2005, the MCL was established to encourage physical activity, sportsmanship, and social cohesion among women from diverse backgrounds in Israel [28,29,30,31,32]. The league has gained significant traction, with thousands of participants from over 90 Jewish and Arab municipalities regularly competing in local and national tournaments [31,32,33]. Beyond sports, MCL members engage in family-oriented activities and community events that strengthen social ties and promote belonging [28,31,32,33].

The novelty and uniqueness of this study lie in its focus on evaluating a culturally tailored, community-based intervention specifically designed for Arab women, a population that often faces significant social and cultural barriers to health promotion. Unlike many health interventions that address only one aspect of well-being, the Mamanet Cachibol League adopts a holistic approach by integrating physical activity, mental health support, leadership development, and community engagement into a single program. This study is unique in not only assessing the program’s impact on multiple health dimensions—such as perceived health, mental health, and lifestyle behaviors—but also in identifying the sociodemographic factors that influence its effectiveness. Moreover, its quasi-experimental design allowed for real-world application and ecological validity while addressing the practical and ethical challenges of randomization. By combining these elements, this research contributes new insights into the role of culturally sensitive sports programs in reducing health disparities and promoting health equity among minority and underserved populations, offering a scalable model for similar contexts worldwide.

Given the importance of sports in promoting women’s health [29,30,31,32,33], particularly among minority populations, this study explores the impact of the MCL program on Arab women participants. The primary objective of this study is to evaluate the effectiveness of the Mamanet Cachibol League program in improving health outcomes among Arab women. Specifically, it aims to assess changes in perceived health status, mental health (subjective happiness, depression, and social capital), and healthy lifestyle behaviors (physical activity and nutrition) following participation in the program. Additionally, this study seeks to explore the influence of sociodemographic factors on these outcomes, contributing to the development of culturally sensitive health promotion strategies. The following hypotheses were formulated: 1. Mental Health: Participation in the MCL would lead to improvements in subjective happiness and reductions in depressive symptoms compared to the control group. 2. Social Capital: Participants in the MCL would experience increased social capital, characterized by greater trust, support, and community engagement, over time. 3. Healthy Lifestyle Behaviors: Engagement in the MCL program would result in increased physical activity levels and better adherence to healthy nutritional practices compared to the control group. 4. Health Perception: Participation in the MCL would positively influence participants’ self-reported health status compared to non-participants.

## 2. Materials and Methods

### 2.1. Study Setting and Description of the Mamanet Cachibol League Intervention Program (MCLIP)

The MCLIP was created as a collaborative effort by several organizations: Ariel University, the Israel Ministry of Health, the Israel Ministry of Sport, Clalit Health Services, and the Israeli Mamanet Cachibol League. The program took place in northern Israel. The MCLIP was conducted to promote physical activity and community engagement and to empower Arab women in Israel. These programs focus on enhancing physical fitness, social cohesion, healthy lifestyle awareness, and leadership skills. The participants met at the sports center once a week for practice and once a week for games and tournaments; the intervention program took place with activities focusing on the Cachibol game, and sessions on nutrition and coaching were led by experts: a dietician, two mental coachers, and three researchers. Additional activities were related to group dynamics and developing personal and group leadership. Women were chosen non-randomly to participate in the program. Each activity lasted about 90 min and was led by a coach who guided the women in the activity at the sports hall. Two additional sessions were held; the first was a mental coaching program for all team captains to enhance leadership skills, and the second was for all participants to learn about healthy nutrition. The program took place between November 2023 and August 2024.

### 2.2. Research Design

This research employed a quasi-experimental design to evaluate the impact of the MCL program on Arab women’s health outcomes and lifestyle habits. It examined two groups of mothers: those who participated in the MCL and those who did not participate in any organized sports (non-participant control group). Data were collected at two different time points. The women were asked to answer the study questionnaire (which included 68 questions) during the first two months of joining the league (T1, the end of 2023 to the beginning of 2024) and 10–11 months later (T2, July–August 2024). The participants of the control group were asked to fill in the study questionnaire at the same time point. The sample of Mamanet players was recruited with the help of the Mamanet organization; the organization provided the names and phone numbers of the captains, to whom the questionnaire was sent. The captains sent out an online questionnaire via WhatsApp. Each team has a WhatsApp group by which they communicate, run by the captain. There was no interaction between the intervention and control groups. Those who responded to the questionnaire at T1 were sent another request to complete the questionnaire at T2.

The non-participant control group was recruited from an Internet panel of 100,000 members. An online questionnaire was sent out to a random sample of women aged 30–50 with at least one child between the ages of 6 and 18. These women reported not participating in organized competitive sports like the Mamanet League. Those who responded at T1 answered the questionnaire again at T2. They answered the questionnaires at the same time as the Mamanet players.

The ethics committee of the Faculty of Health Sciences, Ariel University, approved this study. All respondents were given explanations before data collection and were advised that participation was voluntary. The number of the ethics committee is AU-HEA-RT-20221225.

**Figure 1:**
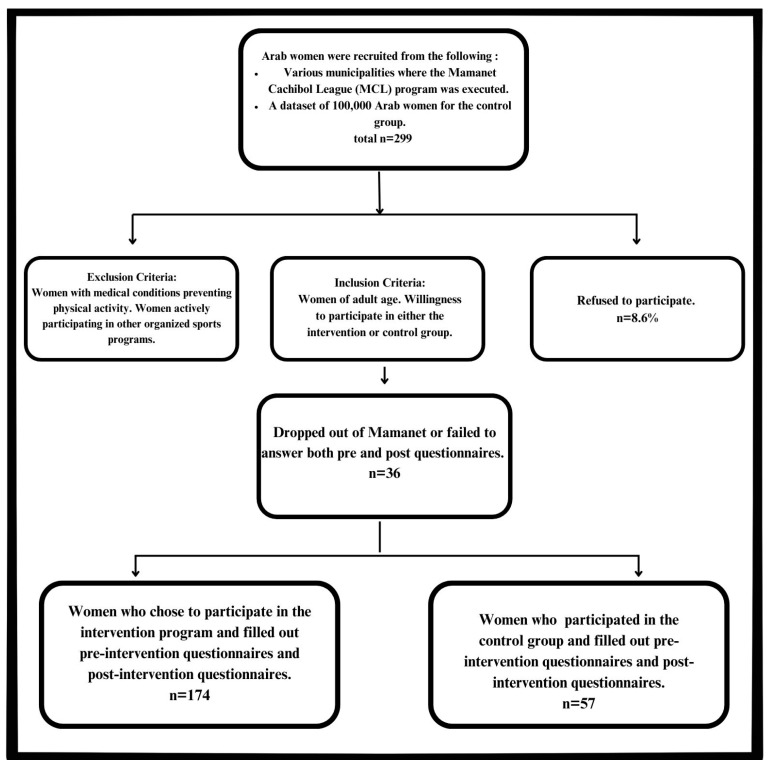
Study population progression.

A total of 231 participants involved in the MCL were eligible for the intervention and received an explanation about the program. A total of 20 women refused to participate, leaving 211 who agreed to consent and filled out T1 questionnaires. Overall, 25 of the participants did not partake in T2 and thus dropped out, yielding 186 participants (11.8% attrition). However, we could not match the T1 and T2 questionnaires for 12 participants, yielding a total sample size of 174 participants; this amounted to a 17.5% loss to follow-up. The control group included 68 respondents at T1 and 57 respondents at T2; the loss to follow-up was 16.1%. An analysis was conducted on the data of the 57 women who answered both the T1 and the T2 questionnaires. We compared the respondents and those lost to follow-up and found no significant difference between the groups in age, number of children, marital status, religiosity, and education. For further details, see Figure 1 (Study population progression).

### 2.3. Measures and Instruments

The analysis included several key variables. Each questionnaire contained 100 questions covering the participants’ sociodemographic characteristics and six dependent variables: health perception, mental health (assessed through subjective happiness, depression, and social capital), and a healthy lifestyle (focused on physical activity and nutrition). All variables were measured at both the start and end of this study. All materials were provided in both Hebrew and Arabic, ensuring accessibility for participants from different linguistic backgrounds. Where necessary, validated versions of the instruments in these languages were utilized. Tools such as the Subjective Happiness Scale (SHS), Patient Health Questionnaire (PHQ-9), International Physical Activity Questionnaire (IPAQ), and the Mediterranean Diet Adherence Screener (MEDAS) have been validated in Hebrew or Arabic, ensuring their cultural and linguistic appropriateness. These translated instruments were taken from the Israeli Ministry of Health.

#### 2.3.1. Sociodemographic Characteristics

Sociodemographic characteristics included age, number of children, family and marital status, religiosity (secular or religious), education level (academic or non-academic), and current occupational status [34].

#### 2.3.2. Health Status

Health status was evaluated using self-reported health (SRH), which consists of the standard question: “How do you evaluate your health generally?” Answers range between 1 and 6, with 1 = excellent and 6 = very bad [30].

#### 2.3.3. Mental Health

Subjective Happiness Scale (SHS)—This scale assessed participants’ mental well-being through four items, with the fourth being reverse scored. Scores range from 1 to 7, with higher scores indicating greater subjective happiness. While there is no definitive cut-off point, scores of 6 or higher indicate above-average happiness, while scores below 3.5 suggest below-average happiness. The typical range in American populations is 4.5 to 5.5 (SD 1) [35].

Depression Patient Health Questionnaire (PHQ 9)—This validated tool measures psychological distress and depression levels using nine items. The sum scores are interpreted as follows: 0–5, No depression; 5–9, Mild depression; 10–14, Moderate depression; 15–19, Moderately severe depression; and 20–27, Severe depression [36].

Social capital—The social capital measure consisted of 13 questions about social support, trust, and social involvement. The social support domain included five items, such as “How many close friends do you have?” and “To what extent do you feel appreciated by society?” The trust domain included three items such as “In general, do you think that most people can be trusted, or that you can’t be too careful in dealing with people?” and “In your opinion, would most people try to take advantage of you if given a chance, or would they try to be fair to you?” Finally, the social involvement domain included four items: “To what extent have you participated in any community event in the past six months?” and “To what extent are you likely to meet friends or acquaintances when you go shopping in your area of residence?”. Each question is scored on a scale of 0 to 10, with higher scores representing greater social capital. Inter-correlations between the three sub-scales were significant and ranged between r = 0.25 and r = 0.49 (*p* < 0.001), and thus, a total score for social capital was composed and used in the analysis (α = 0.79 for T1 and 0.84 for T2) [30,37].

#### 2.3.4. Healthy Lifestyle

Physical activity—The International Physical Activity Questionnaire was used to establish physical activity levels. This questionnaire quantifies physical activity performed by the individual in the preceding seven days. Subjects are scored based on the number of days per week and duration of time per day spent undertaking vigorous-intensity activity, such as aerobics, and moderate-intensity activity, such as leisure cycling and walking. For the purpose of this study, the number of weekly minutes engaged in moderate and vigorous aerobic physical activity was calculated [38].

Nutrition—Nutrition was evaluated using a validated 17-item questionnaire designed to assess adherence to the Mediterranean diet. The scoring method awards 1 point for each criterion met and 0 points if the criterion is not met. For instance, an individual consuming less than one vegetable per day would receive a score of 0 for daily vegetable intake. The total score ranges from 0 to 17, with higher scores indicating greater adherence to the Mediterranean diet principles [39,40].

### 2.4. Data Analysis

The normality distribution of the main continuous variables was performed using Q-Q plots. As all main variables were normally distributed, parametric statistics were used.

#### 2.4.1. Sociodemographic Characteristics of Study Participants

The participants’ sociodemographic characteristics were analyzed using descriptive statistics, including mean, standard deviation, frequency (n), and percentage (%). Comparisons between the intervention and control groups in sociodemographic factors were conducted using independent *t*-tests for continuous variables and chi-square tests for categorical variables.

#### 2.4.2. Between- and Within-Group Differences and Changes in Health and Healthy Lifestyle Characteristics in the Intervention Group vs. the Control Group

Between-group differences in health and lifestyle characteristics at the pre-test and post-test were evaluated using independent *t*-tests. Changes from the pre-test to the post-test in each study group were assessed using paired *t*-tests. In addition, Cohen’s d-effect size (ES; mean ∆/standard deviation average from two means) was calculated in order to examine the extent of changes from the pre-test to the post-test. A correction for the dependence among means was conducted using the correlations between the two means following Morris and DeShon’s equation [41]. Generally, values <0.20 were considered as trivial effect sizes, between 0.20 and 0.50 as small effect sizes, 0.51 and 0.80 as moderate effect sizes, and >0.80 as large effect sizes [42].

#### 2.4.3. Health and Lifestyle Differences Among Intervention Group Participants Based on Sociodemographic Characteristics

Within the intervention group, the relationships between continuous sociodemographic variables (age and number of children) and health outcomes and healthy lifestyle habits were assessed using Pearson correlation analyses. Differences in health and lifestyle characteristics across various categorical sociodemographic variables were evaluated using chi-square tests.

#### 2.4.4. Pre-to-Post-Test Changes in Health and Lifestyle by Sociodemographic Characteristics—Intervention Group

Changes in health and healthy lifestyle characteristics from the pre-test to the post-test, based on various categorical sociodemographic factors, were analyzed using paired *t*-tests. Effect sizes were calculated to assess the magnitude of these changes.

SPSS Statistics for Windows, version 29 [43], was used for all statistical analyses. The level of significance was set at *p* < 0.05 (2-tailed). Only power analysis was conducted using G*Power software version 3.1.9.2.2.6.

## 3. Results

### 3.1. Sociodemographic Characteristics of the Study Participants

The intervention and control groups differed significantly across various sociodemographic factors. For instance, compared to the control group, the participants in the intervention group were older (mean age: 43.1 ± 5.7 years in the intervention group vs. 40.1 ± 5.9 years in the control group; t = −3.37, *p* = 0.001), had more children (mean number of children: 3.1 ± 1.2 in the intervention group vs. 2.5 ± 1.0 in the control group; t = −3.28, *p* = 0.001), and had lower education levels (19.5% of the intervention group vs. 50.9% of the control group had a Bachelor’s degree; χ^2^ = 21.26, *p* < 0.001). For further details, see Table 1.

### 3.2. Comparisons Between Groups

**Figure 2:**
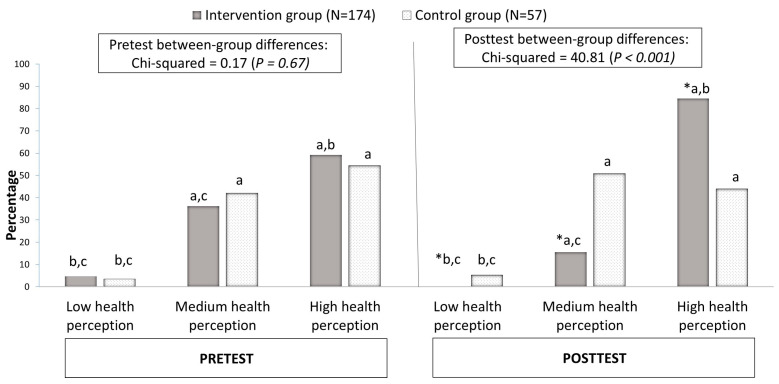
Between- and within-group differences and changes in general health status before and after the intervention. Notes: * between-group differences: prevalence in the “control group” is significantly different from the prevalence in “intervention group” (*p* < 0.05); a, within-group differences: significantly different from the prevalence of low health perception (“very bad” and “not god”) (*p* < 0.05); b, within-group differences: significantly different from the prevalence of medium health perception (“reasonable” and “d”) (*p* < 0.05); c, within-group differences: significantly different from the prevalence of high health perception (“very good” and “excellent”) (*p* < 0.05).

Both study groups demonstrated similar physical health status and general health perception at the pre-test stage. In the post-test, the participants in the intervention group exhibited a more favorable general health status, with 90% reporting a high perception of their health compared to 48% in the control group. Improvements in physical activity and dietary adherence were also more pronounced in the intervention group compared to the control group. The intervention group’s positive outcomes were significantly greater than those of the control group, indicating the effectiveness of the Mamanet Cachibol League in promoting mental and physical health. For further details, see Figure 2 (general health status results).

Regarding mental health, the intervention group outperformed the control group in all assessed variables at the pre-test and post-test stages. Specifically, the participants in the intervention group reported higher subjective happiness, lower levels of depression, and greater social capital (see Table 2).

Regarding healthy lifestyle characteristics, physical activity levels were similar between the two groups at both the pre-test and the post-test. However, the nutrition of the participants in the intervention group was superior to that of the participants in the control group at both stages (see Table 2).

### 3.3. Health Outcomes in the Intervention and Control Groups

The proportion of the participants reporting high health perception significantly increased from 65% to 90% (*p* < 0.05; see Figure 2). Overall, the intervention group demonstrated significant improvements in subjective happiness, reductions in depressive symptoms, and enhanced social capital over the study period. Conversely, the control group showed a decline in some outcomes, including increased depressive symptoms. These negative trends suggest a possible deterioration in mental health among the non-participants, which may underscore the protective role of structured community interventions like the MCL.

In terms of mental health, the control group showed a significant change in only one outcome—depression—though the change was negative, indicating a significant increase in depressive symptoms. The ES associated with this change was trivial (ES = 0.15). In contrast, the intervention group showed positive changes across all three mental health measures. Specifically, the intervention group demonstrated a significant increase in subjective happiness and a significant decrease in depression, with moderate ESs (ES = 0.45 for subjective happiness and ES = −0.22 for depression). While the participants in the intervention group also showed increased social capital, the ES for this change was trivial (ES = 0.11; see Table 2).

Finally, regarding healthy lifestyle characteristics, the control group showed no significant changes in the two variables studied (physical activity level and nutrition). In contrast, the intervention group significantly improved both their physical activity levels and nutrition, with a moderate ES observed for nutrition (ES = 0.25; see Table 2).

### 3.4. Differences in Health and Healthy Lifestyle Between Participants with Different Sociodemographic Characteristics—Intervention Group

In the next phase, the participants’ health and lifestyle outcomes were analyzed based on their sociodemographic traits to understand how sociodemographic characteristics influence health and healthy lifestyles within the intervention group. The analysis revealed that the participants in a relationship had higher physical activity levels at both the pre-test and the post-test compared to those not in a relationship. Additionally, the participants with lower education levels demonstrated lower social capital than those with higher education in both the pre-test and the post-test. Lastly, the unemployed participants (including homemakers) had lower social capital at both the pre-test and the post-test compared to the employed and self-employed individuals. For further details, see Table 3.

### 3.5. Changes in Health and Healthy Lifestyle According to Sociodemographic Characteristics—Intervention Group

In four out of the seven health and healthy lifestyle outcomes assessed, moderate to large effect sizes from the pre-test to the post-test were observed across most participants, regardless of their sociodemographic characteristics. Specifically, in health perception, all sociodemographic groups demonstrated moderate to large effect sizes (effect size range: 0.27 for the participants with a graduate degree to 0.85 for the participants with a high school degree). Similar results were found for subjective happiness, with an effect size ranging from 0.27 among the religious participants to 0.76 among those not in a relationship. Additionally, for both depression and nutrition, most participants, irrespective of sociodemographic traits, exhibited moderate to large effect sizes. In contrast, only a limited number of subgroups within the intervention group participants displayed moderate to large effect sizes on social capital and physical activity. For further details, refer to Table 4.

An interesting observation is that the participants in a relationship showed greater effect sizes compared to those not in a relationship, particularly in health perception and nutrition. Conversely, the opposite trend was noted for subjective happiness, where those not in a relationship had larger effect sizes. Furthermore, the participants with higher levels of education, particularly those with a graduate degree, exhibited smaller effect sizes across all health and healthy lifestyle outcomes compared to those with lower education levels. Similarly, Muslims showed larger effect sizes than non-Muslims in health perception (Table 4). Finally, associations between continuous sociodemographic characteristics and health and healthy lifestyle outcomes were explored. The analysis revealed that the number of children was positively associated with physical activity levels (r = 0.15, *p* = 0.03).

## 4. Discussion

The current study examined the impact of the MCL program on Arab women participants. Specifically, we explored whether the participants in the MCL have higher levels of subjective happiness, social capital, healthy lifestyle (physical activity and nutrition), and low levels of depression symptoms compared with the control group and whether these components improve over time.

Our findings suggest that women participating in the MCL have higher subjective happiness, social capital, and healthy lifestyle levels than those not participating in organized sports (the control group). Our results showed that participation in the Mamanet program yielded significant improvements in subjective happiness and reductions in depressive symptoms among Arab women. These results are consistent with previous research indicating the psychological benefits of regular physical activity and social engagement [30,31,32]. The observed increase in subjective happiness can be attributed to the supportive environment fostered by the MCL, where women experience a sense of belonging and companionship.

Furthermore, the reduction in depression symptoms highlights the mental health benefits of structured, community-based physical activity, reinforcing the value of programs that integrate physical and social well-being components [26,29]. Moreover, this study demonstrates that the MCL participants experienced a marked increase in social capital, underscoring the importance of social networks in enhancing overall well-being. The MCL’s emphasis on team-based activities and community involvement allows women to build trust, form meaningful connections, and engage in collective endeavors. These aspects go along with previous results about team sport participation, leading to the participants’ compliance to, engagement in, commitment to, and continuation of the involvement in team sport for well-being purposes [45,46]. Social interactions are crucial, especially in traditional communities, where women may face cultural restrictions that limit their social engagement. The increase in social capital observed in this study suggests that the MCL successfully addresses these barriers, fostering a more cohesive and supportive community. This result is supported by previous results implying that participating in sports is related to higher social connectedness over time and is beneficial for several social outcomes, including prosocial behavior, interpersonal communication, and fostering a sense of belonging [2,46].

The MCLIP was also associated with notable improvements in physical activity and nutrition. The participants reported higher levels of physical activity and better adherence to healthy dietary practices, which are critical for long-term health [47]. These changes were moderate in effect size, indicating meaningful improvements that could contribute to the prevention of chronic health conditions prevalent among Arab women, such as obesity and cardiovascular disease [22,23,24,48]. The program’s integration of nutrition education and physical coaching likely played a pivotal role in these outcomes, emphasizing the need for comprehensive, multifaceted health promotion strategies.

The study results also highlight the influence of sociodemographic factors on health and lifestyle outcomes. Older age and having more children were associated with greater participation and positive health behaviors, suggesting that women at different life stages may experience varying benefits from the MCL. Moreover, the higher age of the participants in the MCL might indicate that older participants have more time or resources to dedicate to such activities compared to younger women, who might still be in the earlier, more demanding phases of their careers or motherhood. Having more children may indicate women’s motivation to engage in programs like the MCL, potentially to model active lifestyles for their children, or to use these activities as a social and health resource amidst their parenting responsibilities. This result may also be explained by other findings, suggesting that team sport was used by mothers to exercise their identities as athletes. Doing so allowed them to balance their two identities, feel more rounded and confident in other aspects of their lives, and add a dimension to their identities, defining them as more than just mothers [48,49,50,51].

Another critical sociodemographic aspect is education level. While 50.9% of the control group held a Bachelor’s degree, only 19.5% of the intervention group did. This significant disparity suggests that MCL participants prioritize and benefit significantly from program participation despite their lower educational attainment. This aligns with [52,53], who emphasize that the social capital cultivated through the MCL can enhance well-being irrespective of educational background. The lower educational level among the intervention group participants underscores the value of social and community-based programs like the MCL as vital resources for health promotion across diverse academic backgrounds.

The worsening outcomes in the control group, particularly the increase in depressive symptoms, highlight the potential risks of social isolation and lack of structured physical activity. These results align with international findings that underscore the importance of structured interventions in mitigating mental health challenges in under-represented populations. Factors such as limited access to health resources and increased psychosocial stressors may have contributed to these negative trends. This contrast underscores the protective benefits of the Mamanet program, which provided participants with opportunities for social engagement, physical activity, and skill-building.

Previous studies have demonstrated similar outcomes. Research on community sports programs in the United States has shown that structured group activities can improve social connectedness and reduce depressive symptoms, particularly among women from underserved populations. Studies conducted in Europe have highlighted the role of team sports in promoting physical activity adherence and reducing sedentary lifestyles in women, aligning closely with the outcomes observed in our study [54]. Additionally, research in the Middle East has shown that culturally tailored interventions, like those emphasizing community cohesion and inclusivity, significantly enhance participants’ mental health and dietary behaviors [55]. These findings reinforce the universality of the benefits associated with community-based sports programs and validate the Mamanet Cachibol League as an effective approach for addressing health disparities in minority populations.

## 5. Practical Implications and Future Research

The MCL model’s success lies in its culturally sensitive approach, which respects and integrates the values and traditions of Arab communities. The program addresses cultural barriers that have historically limited women’s participation in sports by providing a safe and supportive environment for women to engage in physical activity. The significant health improvements reported in this study suggest that culturally tailored interventions are crucial for promoting physical activity in conservative societies.

Hence, it is recommended that public health policymakers and practitioners draw upon these findings to develop and implement similar programs tailored to the specific needs of diverse populations. Additionally, healthcare providers can incorporate information about the benefits of community-based sports into their clinical practice, encouraging patients to participate in such activities. Future research should investigate long-term outcomes and identify strategies to extend these benefits to broader community settings. Understanding the contextual factors contributing to the control group’s negative trends will be crucial for refining intervention designs and addressing systemic barriers to health equity.

## 6. Limitations

While this study provides valuable insights into the impact of the MCL program on Arab women’s health, certain limitations should be acknowledged. Firstly, the self-reported nature of the data may introduce potential biases, as the participants may have overestimated or underestimated their adherence to healthy behaviors. Additionally, the relatively small sample size and specific demographic characteristics of the participants may limit the generalizability of the findings to other populations.

An additional limitation of this study is the lack of randomization in the experimental group. Unlike the control group, which was randomized, the participants in the intervention group voluntarily enrolled in the Mamanet Cachibol League. This introduces the possibility that the observed differences between the groups may partially reflect pre-existing factors, such as a greater motivation to exercise or other psychosocial characteristics of the participants. While steps were taken to mitigate these biases, such as using a matched control group and controlling for confounding variables, the absence of randomization remains a potential source of bias. Future studies should consider employing randomized controlled trials or other methods to further validate these findings.

## 7. Conclusions

This study provides compelling evidence of the MCL’s effectiveness in enhancing Arab women’s physical and mental health through culturally tailored, community-based interventions. By fostering physical activity, reducing depressive symptoms, and promoting community engagement, the MCL program has demonstrated its potential as a scalable and replicable model for addressing health disparities. Unlike traditional health interventions, the program’s holistic approach integrates leadership development and social support, reinforcing the protective effects of community and teamwork. The MCL exemplifies how community-based interventions can empower women to take charge of their well-being while simultaneously addressing broader systemic barriers to health equity. Policymakers and health practitioners should consider implementing and scaling similar interventions in other culturally diverse settings to promote sustainable health improvements and reduce disparities. Ultimately, this study reinforces the value of culturally tailored, community-focused health promotion initiatives in fostering resilience, well-being, and social cohesion on a global scale.

## Figures and Tables

**Table 1:** Sociodemographic characteristics of the study participants.

Characteristics		Intervention Group (n = 174)	Control Group (n = 57)	Between-Group Differences: t Score (*p*-Value) or Chi-Square (*p*-Value)
Age, years: mean (SD)		43.1 (5.7)	40.1 (5.9)	0.001
Family status: n (%)	Single	14.0 (8.0)	8.0 (14.0)	0.18
	In a relationship	150.0 (86.2)	47.0 (82.5)	0.46
	Divorced	3.0 (1.7)	1.0 (1.8)	0.95
	Widow	7.0 (4.0)	1.0 (1.8)	0.43
Children, number: mean (SD)		3.1 (1.2)	2.5 (1.0)	0.001
Education: n (%)	High school	49.0 (28.1)	8.0 (14.0)	0.03
	Professional certificate	56.0 (32.2)	11.0 (19.3)	0.06
	Bachelor’s degree	34.0 (19.5)	29.0 (50.9)	<0.001
	Graduate degree	35.0 (20.1)	9.0 (15.8)	0.47
Religion: n (%)	Arab—Christian	36.0 (20.6)	11.0 (19.3)	0.83
	Arab—Muslim	131.0 (75.3)	34.0 (59.6)	0.02
	Druze	3.0 (1.7)	11.0 (19.3)	<0.001
	Other	5.0 (2.8)	1.0 (1.8)	0.67
Religiosity level: n (%)	Secular	56.0 (32.1)	12.0 (21.1)	0.11
	Traditional	34.0 (19.5)	29.0 (50.9)	<0.001
	Religious	54.0 (31.0)	14.0 (24.6)	0.35
	Orthodox	12.0 (6.9)	-	0.04
	Refuse or unknown	18.0 (10.3)	2.0 (3.5)	0.11
Employment status: n (%)	Employee	94.0 (54.0)	43.0 (75.4)	0.004
	Self-employed	30.0 (17.2)	4.0 (7.0)	0.05
	Unemployed	4.0 (2.3)	1.0 (1.8)	0.82
	Housewife	28.0 (16.1)	3.0 (5.3)	0.03
	Other	18.0 (10.3)	6.0 (10.5)	0.96

Notes. SD, standard deviation.

**Table 2:** Between- and within-group differences and changes in mental health status and healthy lifestyle characteristics—intervention group vs. control group.

Assessment Domain	Scale’s Name	Intervention Group (n = 174)			Control Group (n = 57)			Between-Group Differences	
		Pre-Test: Mean (SD)	Post-Test: Mean (SD)	Within-Group Changes: t (*p*-Value) [ES]	Pre-Test: Mean (SD)	Post-Test: Mean (SD)	Within-Group Changes: t (*p*-Value) [ES]	Pre-Test: t (*p*-Value)	Post-Test: t (*p*-Value)
Mental health	Subjective Happiness Scale, mean score	5.0 (1.2)	5.6 (0.8)	<0.01	4.6 (1.1)	4.5 (1.1)	0.06	−2.40 (0.01)	<0.01
	Depression, sum score	5.0 (4.4)	4.0 (4.0)	−2.35 (0.01) [−0.22]	8.4 (5.3)	9.3 (3.9)	2.30 (0.02) [0.15]	4.40 (<0.01)	8.75 (<0.001)
	Social capital, sum scores	50.00 (13.7)	52.5 (14.4)	3.66 (<0.001) [0.11]	45.0 (11.4)	45.2 (10.7)	0.56 (0.57) [0.01]	−3.21 (<0.001)	−4.06 (<0.001)
Healthy lifestyle characteristics	Physical activity, weekly minutes in moderate and vigorous aerobic physical activity	221.3 (437.2)	248.2 (438.3)	3.73 (<0.001) [0.06]	206.5 (128.6)	204.0 (191.0)	−0.32 (0.74) [−0.05]	−0.39 (0.69)	−1.03 (0.30)
	Nutrition, IMED sum score	8.0 (1.3)	8.4 (1.4)	3.92 (<0.001) [0.25]	7.2 (1.7)	7.4 (1.6)	0.83 (0.40) [0.07]	−3.06 (0.003)	−3.96 (<0.001)

**Table 3:** Differences in health and healthy lifestyle according to sociodemographic characteristics—intervention group.

	Health		Happiness		Depression		Social Capital		Physical Activity		Nutrition	
	Pre	Post	Pre	Post	Pre	Post	Pre	Post	Pre	Post	Pre	Post
Not in a relationship (n = 24)	4.6 (1.0)	5.0 (0.8)	4.8 (1.0)	5.6 (0.8)	5.0 (6.2)	3.0 (3.0)	55.2 (14.0)	56.9 (14.7)	196.6 (184.4)	191.2 (173.5)	8.0 (1.4)	8.1 (1.8)
Relationship (n = 149)	4.6 (1.08)	5.2 (0.7)	5.1 (1.2)	5.6 (0.8)	5.0 (4.0)	4.1 (4.1)	50.3 (13.5)	51.8 (14.3)	225.2 (465.4) *	257.3 (466.7) *	8.0 (1.3)	8.4 (1.4)
High school (n = 47) ^a^	4.3 (1.0)	5.2 (0.7)	5.1 (1.2)	5.8 (0.7)	5.4 (4.4)	2.9 (3.3)	45.8 (15.1) ^b,c,d^	47.1 (16.3) ^c^	323.9 (796.9)	344.6 (799.1)	7.8 (1.2)	8.3 (1.6)
Professional certificate (n = 56) ^b^	4.6 (1.0)	5.2 (0.7)	4.9 (1.2)	5.5 (0.9)	5.4 (5.1)	4.1 (3.8)	50.9 (12.8) ^a^	52.3 (13.3)	202.9 (175.6)	218.8 (174.7)	8.1 (1.3)	8.7 (1.3)
Bachelor’s degree (n = 33) ^c^	4.6 (1.0)	5.3 (0.6)	5.2 (1.0)	5.7 (0.7)	3.1 (3.0)	4.6 (3.9)	54.4 (13.0) ^a^	57.5 (13.7) ^a^	161.6 (131.4)	227.4 (148.7)	8.3 (1.2)	8.2 (1.5)
Graduate degree (n = 35) ^d^	4.8 (1.1)	5.2 (0.7)	5.0 (1.1)	5.4 (0.9)	5.4 (4.0)	4.6 (4.6)	54.4 (12.0) ^a^	54.9 (12.1)	181.7 (144.9)	198.3 (150.9)	7.8 (1.4)	8.0 (1.5)
Not Muslim (n = 39)	4.7 (1.3)	5.2 (0.6)	5.6 (0.7)	5.8 (0.7)	4.7 (3.8)	4.0 (3.9)	55.1 (11.7)	55.7 (11.2)	155.3 (151.5)	170.7 (151.8)	8.1 (1.1)	8.4 (1.1)
Muslim (n = 130)	4.2 (1.2) *	5.2 (0.7)	5.2 (1.1) *	5.5 (0.9)	4.7 (3.8)	3.9 (4.0)	47.9 (13.7)	52.2 (14.6)	194.6 (109.7)	274.4 (495.6)	7.3 (1.0)	8.3 (1.5)
Secular (n = 56)	4.8 (0.9)	5.3 (0.7)	5.1 (1.1)	5.9 (0.7)	4.8 (5.1)	4.3 (4.2)	52.8 (13.1)	54.6 (14.4)	283.5 (736.4)	317.2 (730.8)	8.3 (1.1)	8.6 (1.5)
Traditional (n = 34)	4.6 (1.1)	5.2 (0.7)	4.9 (1.1)	5.4 (0.8)	5.0 (4.3)	4.7 (5.0)	50.1 (15.8)	52.0 (17.0)	199.9 (153.5)	214.5 (149.9)	8.0 (1.4)	8.2 (1.5)
Religious (n = 53)	4.5 (0.9)	5.2 (0.6)	5.0 (1.3)	5.4 (1.0)	4.9 (4.0)	3.4 (3.4)	51.5 (13.7)	52.4 (13.7)	183.0 (169.8)	211.0 (201.2)	8.0 (1.4)	8.2 (1.3)
Orthodox (n = 12)	4.4 (1.0)	4.7 (0.7)	5.3 (0.9)	5.8 (0.8)	6.1 (3.7)	4.5 (4.1)	46.3 (8.7)	49.5 (8.5)	204.08 (183.4)	230.0 (185.9)	7.9 (1.3)	8.5 (1.2)
Refuse (n = 18)	4.2 (1.2)	5.11 (0.7)	5.2 (1.1)	5.6 (0.7)	4.7 (3.8)	2.7 (2.0)	47.9 (13.7)	49.3 (14.7)	194.6 (109.7_	220.5 (111.6)	7.3 (1.0)	8.1 (15)
Employee (n = 94) ^e^	4.7 (1.0)	5.2 (0.6)	5.1 (1.0)	5.6 (0.8)	4.7 (4.1)	4.5 (4.6)	51.5 (12.8) ^g^	53.1 (13.7) ^g^	214.3 (174.5)	238.9 (170.3)	8.0 (1.3)	8.2 (1.4)
Self-employed (n = 29) ^f^	4.5 (1.0)	5.4 (0.6)	5.1 (1.0)	5.5 (0.8)	4.3 (4.8)	2.9 (2.9)	56.7 (9.7) ^g^	57.7 (10.1) ^g^	132.7 (126.5)	182.1 (153.8)	8.1 (1.3)	8.6 (1.4)
Unemployed (n = 32) ^g^	4.6 (0.9)	5.2 (0.7)	5.0 (1.5)	5.7 (1.0)	5.9 (4.8)	4.0 (3.3)	43.7 (15.7) ^e,f,h^	44.9 (16.9) ^e,f,h^	332.6 (964.9)	352.0 (968.7)	8.1 (1.2)	8.4 (1.4)
Other (n = 18) ^h^	4.2 (1.1)	4.8 (0.8)	5.0 (1.2)	5.7 (0.7)	5.8 (4.4)	3.0 (2.5)	51.5 (15.3) ^g^	54.4 (15.1)^g^	207.3 (123.3)	221.9 (123.7)	7.7 (1.2)	8.6 (1.6)

Notes: * significant between-categorical-group differences (*p* < 0.05); ^a^ significantly different from “high school” (*p* < 0.05); ^b^ significantly different from “Professional certificate” (*p* < 0.05); ^c^ significantly different from “Bachelor’s degree” (*p* < 0.05); ^d^ significantly different from “graduate degree” (*p* < 0.05); ^e^ significantly different from “Employee” (*p* < 0.05); ^f^ significantly different from “self-employed” (*p* < 0.05); ^g^ significantly different from “unemployed/housewife” (*p* < 0.05); ^h^ significantly different from “other” (*p* < 0.05); SD, standard deviation.

**Table 4:** Changes in health and healthy lifestyle according to sociodemographic characteristics—intervention group.

Sociodemographic Characteristics		Health Perception: Effect Size	Subjective Happiness: Effect Size	Depression: Effect Size	Social Capital: Effect Size	Physical Activity: Effect Size	Nutrition: Effect Size
Family status	Not in a relationship (n = 24)	0.37 *	0.76 *	−0.31 *	0.12 *	−0.02	0.08
	In a relationship (n = 149)	0.60 *	0.41 *	−0.20 *	0.11 *	0.06	0.28 *
Education	High school (n = 47)	0.85 *	0.48 *	−0.55 *	0.08	0.02	0.36 *
	Professional certificate (n = 56)	0.56 *	0.50 *	−0.26 *	0.11 *	0.09	0.40 *
	Bachelor’s degree (n = 33)	0.65 *	0.50 *	0.47 *	0.24 *	0.50 *	−0.04
	Graduate degree (n = 35	0.27 *	0.32 *	−0.19 *	0.04	0.11 *	0.16 *
Religion	Not Muslim (n = 39)	0.35 *	0.26 *	−0.19 *	0.05	0.10 *	0.26 *
	Muslim (n = 130)	0.65 *	0.45 *	−0.19 *	0.13 *	0.06	0.25 *
Religiosity level	Secular (n = 56)	0.59 *	0.69 *	−0.09	0.13 *	0.04	0.29 *
	Traditional (n = 34)	0.44 *	0.46 *	−0.06	0.11 *	0.09	0.14 *
	Religious (n = 53)	0.69 *	0.27 *	−0.37 *	0.06	0.16 *	0.15 *
	Orthodox (n = 12)	0.30 *	0.46 *	−0.44 *	0.37 *	0.14 *	0.42 *
	Refuse/unknown (n = 18)	0.67 *	0.35 *	−0.52 *	0.10 *	0.23 *	0.69 *
Employment status	Employee (n = 94)	0.48 *	0.46 *	−0.05	0.12 *	0.14 *	0.14 *
	Self-employed (n = 29)	0.80 *	0.33 *	−0.28 *	0.09	0.39 *	0.35 *
	Unemployed/housewife (n = 32)	0.69 *	0.43 *	−0.40 *	0.07	0.02	0.25 *
	Other (n = 18)	0.52 *	0.63 *	−0.64 *	0.19 *	0.11 *	0.70 *

Notes: * Correlation is significant at the 0.05 level (2-tailed); ES, effect size. Cells in light gray represent small effect sizes (effect size range: 0.20 and 0.50) and cells in dark gray represent moderate to large effect sizes (effect size > 0.51) [44].

## Data Availability

The datasets used and/or analyzed during the current study are available from the corresponding author upon reasonable request.
